# Supplementary material for: Comprehensive Study of the IBMP ELISA IgA/IgM/IgG COVID-19 Kit for SARS-CoV-2 Antibody Detection
Source: Diagnostics (Basel). 2024 Jul 13;14(14):1514. doi: 10.3390/diagnostics14141514 (PMC11276192; doi:10.3390/diagnostics14141514)
Supplement: Supplementary file 1 [file diagnostics-14-01514-s001.zip › diagnostics-3085268-supplementary.pdf]

Table S1: Reactivity index of SARS-CoV-2-positive and -negative samples using the IBMP ELISA IgA/IgM/IgG COVID-19 kit for SARS-CoV-2 antibody detection.

Sample	Status	Time Post Symptom Onset	Reactivity index (RI)
HMB-2623	SARS-CoV-2-negative sample	Pre-pandemic	0.22
HMB-2624	SARS-CoV-2-negative sample	Pre-pandemic	0.2
HMB-2625	SARS-CoV-2-negative sample	Pre-pandemic	0.22
HMB-2626	SARS-CoV-2-negative sample	Pre-pandemic	0.19
HMB-2627	SARS-CoV-2-negative sample	Pre-pandemic	0.17
HMB-2628	SARS-CoV-2-negative sample	Pre-pandemic	0.19
HMB-2629	SARS-CoV-2-negative sample	Pre-pandemic	0.22
HMB-2630	SARS-CoV-2-negative sample	Pre-pandemic	0.26
HMB-2631	SARS-CoV-2-negative sample	Pre-pandemic	0.2
HMB-2632	SARS-CoV-2-negative sample	Pre-pandemic	0.73
HMB-2633	SARS-CoV-2-negative sample	Pre-pandemic	0.28
HMB-2634	SARS-CoV-2-negative sample	Pre-pandemic	0.27
HMB-2635	SARS-CoV-2-negative sample	Pre-pandemic	0.17
HMB-2636	SARS-CoV-2-negative sample	Pre-pandemic	0.25
HMB-2637	SARS-CoV-2-negative sample	Pre-pandemic	0.24
HMB-2638	SARS-CoV-2-negative sample	Pre-pandemic	0.27
HMB-2639	SARS-CoV-2-negative sample	Pre-pandemic	0.21
HMB-2640	SARS-CoV-2-negative sample	Pre-pandemic	0.22
HMB-2641	SARS-CoV-2-negative sample	Pre-pandemic	0.21
HMB-2642	SARS-CoV-2-negative sample	Pre-pandemic	0.23
HMB-2643	SARS-CoV-2-negative sample	Pre-pandemic	0.26
HMB-2644	SARS-CoV-2-negative sample	Pre-pandemic	0.24
HMB-2645	SARS-CoV-2-negative sample	Pre-pandemic	0.25
HMB-2646	SARS-CoV-2-negative sample	Pre-pandemic	0.23
HMB-2647	SARS-CoV-2-negative sample	Pre-pandemic	0.23
HMB-2648	SARS-CoV-2-negative sample	Pre-pandemic	0.22
LIBCOV_PL-001	SARS-CoV-2-positive sample	0-7 days	0.16
LIBCOV_PL-007	SARS-CoV-2-positive sample	8-15 days	0.23
LIBCOV_PL-020	SARS-CoV-2-positive sample	15-21 days	6.36
LIBCOV_PL-021	SARS-CoV-2-positive sample	8-15 days	4.93
LIBCOV_PL-023	SARS-CoV-2-positive sample	15-21 days	2.82
LIBCOV_PL-026	SARS-CoV-2-positive sample	15-21 days	5.37
LIBCOV_PL-032	SARS-CoV-2-positive sample	8-15 days	0.96
LIBCOV_PL-045	SARS-CoV-2-positive sample	8-15 days	4.15
LIBCOV_PL-056	SARS-CoV-2-positive sample	15-21 days	3.22
LIBCOV_PL-057	SARS-CoV-2-positive sample	15-21 days	4.49
LIBCOV_PL-058	SARS-CoV-2-positive sample	15-21 days	3.62
LIBCOV_PL-082	SARS-CoV-2-positive sample	0-7 days	2.38
LIBCOV_PL-085	SARS-CoV-2-positive sample	8-15 days	5.3
LIBCOV_PL-087	SARS-CoV-2-positive sample	0-7 days	0.18
LIBCOV_PL-093	SARS-CoV-2-positive sample	0-7 days	1.64
LIBCOV_PL-096	SARS-CoV-2-positive sample	15-21 days	4.81
LIBCOV_PL-097	SARS-CoV-2-positive sample	15-21 days	6.58
LIBCOV_PL-098	SARS-CoV-2-positive sample	15-21 days	5.71
LIBCOV_PL-100	SARS-CoV-2-positive sample	8-15 days	2.13
LIBCOV_PL-101	SARS-CoV-2-positive sample	15-21 days	4.99
LIBCOV_PL-102	SARS-CoV-2-positive sample	15-21 days	5.48

LIBCOV_PL-104	SARS-CoV-2-positive sample	15-21 days	6.89	
LIBCOV_PL-107	SARS-CoV-2-positive sample	8-15 days	4.64	
LIBCOV_PL-110	SARS-CoV-2-positive sample	8-15 days		4.83
LIBCOV_PL-124	SARS-CoV-2-positive sample	8-15 days		5.05
LIBCOV_SR-001	SARS-CoV-2-positive sample	0-7 days		1.9
LIBCOV_SR-002	SARS-CoV-2-positive sample	0-7 days		0.17
LIBCOV_SR-009	SARS-CoV-2-positive sample	0-7 days		4.36
LIBCOV_SR-018	SARS-CoV-2-positive sample	15-21 days		4.63
LIBCOV_SR-022	SARS-CoV-2-positive sample	15-21 days		5.79
LIBCOV_SR-027	SARS-CoV-2-positive sample	15-21 days		8.09
LIBCOV_SR-029	SARS-CoV-2-positive sample	15-21 days		4.63
LIBCOV_SR-037	SARS-CoV-2-positive sample	8-15 days		0.62
LIBCOV_SR-039	SARS-CoV-2-positive sample	8-15 days		1.23
LIBCOV_SR-044	SARS-CoV-2-positive sample	8-15 days		5.03
LIBCOV_SR-055	SARS-CoV-2-positive sample	8-15 days		0.34
LIBCOV_SR-061	SARS-CoV-2-positive sample	8-15 days		0.5
LIBCOV_SR-063	SARS-CoV-2-positive sample	15-21 days		6.23
LIBCOV_SR-068	SARS-CoV-2-positive sample	0-7 days		0.31
LIBCOV_SR-073	SARS-CoV-2-positive sample	0-7 days		0.48
LIBCOV_SR-075	SARS-CoV-2-positive sample	8-15 days		1.5
LIBCOV_SR-086	SARS-CoV-2-positive sample	0-7 days		0.2
LIBCOV_SR-090	SARS-CoV-2-positive sample	8-15 days		1.09
LIBCOV_SR-092	SARS-CoV-2-positive sample	8-15 days		2.32
LIBCOV_SR-109	SARS-CoV-2-positive sample	8-15 days		0.42
LIBCOV_SR-111	SARS-CoV-2-positive sample	8-15 days		0.71
LIBCOV_SR-114	SARS-CoV-2-positive sample	8-15 days		0.9
LIBCOV_SR-116	SARS-CoV-2-positive sample	0-7 days		0.2
LIBCOV_SR-120	SARS-CoV-2-positive sample	8-15 days		0.84
LIBCOV_SR-123	SARS-CoV-2-positive sample	15-21 days		2.63
LIBCOV_SR-126	SARS-CoV-2-positive sample	0-7 days		0.17
LIBCOV_SR-133	SARS-CoV-2-positive sample	15-21 days		3.3
LIBCOV_SR-139	SARS-CoV-2-positive sample	0-7 days		2.74
LIBCOV_SR-154	SARS-CoV-2-positive sample	0-7 days		0.43
LIBCOV_SR-162	SARS-CoV-2-positive sample	0-7 days		0.64
LIBCOV_SR-178	SARS-CoV-2-positive sample	15-21 days		0.69
LIBCOV_SR-180	SARS-CoV-2-positive sample	0-7 days		0.17
LIBCOV_SR-188	SARS-CoV-2-positive sample	15-21 days		7.62
LIBCOV_SR-200	SARS-CoV-2-positive sample	0-7 days		0.16
LIBCOV_SR-204	SARS-CoV-2-positive sample	0-7 days		0.19
LIBCOV_SR-207	SARS-CoV-2-positive sample	0-7 days		0.16
LIBCOV_SR-216	SARS-CoV-2-positive sample	0-7 days		0.18
LIBCOV_SR-248	SARS-CoV-2-positive sample	15-21 days		4.73
LIBCOV_SR-273	SARS-CoV-2-positive sample	15-21 days		1.67
